# Peripheral immune cell-derived matrix metalloprotease 8 (MMP8): brain trafficking promotes depression-like behavior

**DOI:** 10.1038/s41392-024-01833-0

**Published:** 2024-05-22

**Authors:** Dietmar Spengler, Theo Rein

**Affiliations:** 1 Max Planck Institute of Psychiatry, Epigenomics of Early-Life Stress, 80804 Munich, Germany; 2 Max Planck Institute of Psychiatry, Molecular Pathways of Depression, 80804 Munich, Germany

**Keywords:** Molecular neuroscience, Molecular medicine, Cellular neuroscience

In a study recently published in *Nature*, Cathomas et al.^1^ report that social stress in mice increases the levels of matrix metalloprotease (MMP)8 in circulating immune cells, thereby altering extracellular space (ECS) and neuronal activity, and ultimately social behavior in susceptible animals. Behavioral deficits resembled those from major depressive disorder (MDD) raising the prospect of MMP8 as a new druggable target in tailored treatments.

MDD has a high and still increasing worldwide prevalence imposing a tremendous burden on patients and societies.^2^ Despite improved insight into the complex interplay between genetic and environmental risk factors in MDD, treatment success remains limited with up to half of the patients not achieving full remission. Psychosocial stress is a well-established risk factor leading to chronic activation of the innate immune system in susceptible individuals. To better understand the molecular mechanisms by which such low-grade inflammation contributes to neuronal function and depression, Cathomas and coworkers used the chronic social defeat stress (CSDS) model.

Inbred mice were exposed to an aggressor mouse over 10 days through physical and sensory contact. Most of the exposed mice developed behavioral impairments resembling major depression, namely social avoidance and anhedonia, and were called stress-susceptible (SUS) mice. Conversely, a subgroup of exposed, yet unaffected mice, were called resilient (RES) mice.

CSDS led to a higher number of circulating proinflammatory Ly6C^hi^ monocytes and neutrophils in both SUS and RES mice. In meninges-free whole brain, however, proinflammatory monocytes increased significantly in SUS mice only. Brain-resident immune cells were unaffected in either group. Differential gene expression was most prominent in circulating inflammatory monocytes from SUS mice and was enriched in genes with a role in innate immunity, inflammation, and ECS.

To answer how circulating monocytes can affect social behavior the authors first sought to clarify where exactly these cells traffic to. Interestingly, whole-brain analysis revealed that monocytes were enriched in the nucleus accumbens (NAc), a brain area essential for the reward system, and correlated strongly with social avoidance. Circulating monocytes are attached locally to blood vessels without invading the perivascular space or the parenchyma.

Brain-trafficked monocytes comprised four unique RNA clusters as evidenced by single-cell sequencing. One of them was enriched in SUS compared to RES mice and featured upregulated genes important for oxidation-reduction, extracellular matrix (ECM), and ECS. Top-ranked genes included *Mmp8*, which was also among the highest-upregulated genes in circulating monocytes. By contrast, monocytes from choroid plexus, dura, or leptomeninges in SUS mice displayed unchanged *Mmp8* expression. Collectively, these results indicate that circulating monocytes are the source of the MMP8 present in the brain.

Increased MMP8 protein expression in the NAc and plasma from SUS mice correlated negatively with social interaction. Likewise, higher amounts of serum MMP8 in depressed patients correlated with perceived stress. Retro-orbitally injected recombinant Mmp8 accessed the NAc parenchyma in SUS mice, raising the question of how Mmp8 passed through the blood–brain barrier (BBB). Previous studies reported enhanced BBB permeability in SUS mice. Here, recombinant Mmp8 accessed the NAc parenchyma only upon experimental weakening of the BBB in combination with subthreshold social defeat, pointing to a complex and yet-to-be-deciphered interplay between these factors.

Intriguingly, increased ECS was observed specifically in the NAc of SUS mice. As protease, MMP8 could break down the ECM, and indeed, ECS was strongly correlated with MMP8 levels. CSDS decreased the levels of aggrecan, an MMP8 substrate and crucial component of neuronal ECS. Moreover, aggrecan levels negatively correlated with serum MMP8, but positively with social interaction. Further strengthening the link between ECS and social behavior, experimental break-down of the ECM in the NAc associated with increased social avoidance.

To establish a causal relationship between MMP8 and stress-evoked social avoidance, the researchers first showed that intraperitoneal injection of Mmp8 coupled with subthreshold social defeat impaired social interaction. Conversely, chimeric mice lacking Mmp8 specifically in peripheral leukocytes exhibited less social avoidance upon CSDS, concomitant with reduced ECS in the NAc. Importantly, other stress-related behaviors including sucrose preference remained unchanged.

Finally, electrophysiological analysis of NAc slices showed that leukocyte-specific depletion of Mmmp8 attenuated stress-dependent increases in excitability and excitatory postsynaptic currents in medium spiny neurons.

Overall, this work highlights a new scale of social stress-dependent interaction between immune and neuronal systems^3^ that will inspire basic and clinical research alike (Fig. 1). For example, it will be important to define pathways and mechanisms by which CSDS leads to, firstly, expansion of peripheral monocytes in SUS and RES mice along with the induction of a SUS-specific transcriptional signature in brain-trafficking monocytes, secondly, site-specific retention of brain-trafficking monocytes at vascular endothelia in the NAc of SUS mice, and thirdly, increases in BBB permeability allowing MMP8 passage into the parenchyma specifically in the NAc (Fig. 1).

**Fig. 1:**
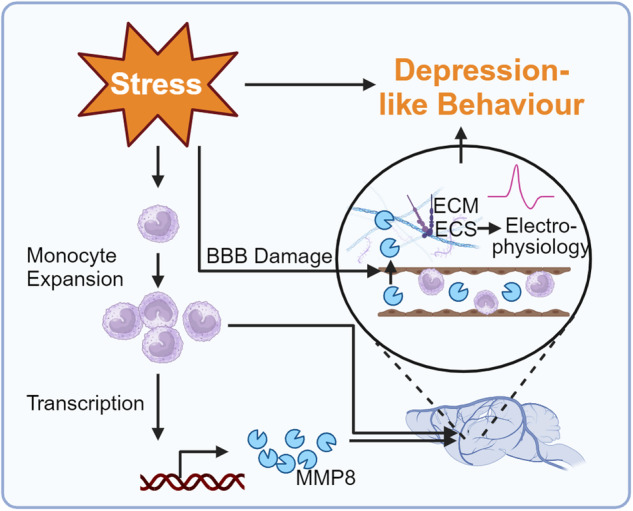
MMP8 links social stress to depression-like behavior and presents a potential drug target. Experience of social stress leads to the expansion of peripheral monocytes including transcription and secretion of MMP8 into the blood flow. Monocytes and MMP8 traffic to the brain, where MMP8 penetrates the pre-damaged BBB in susceptible mice. MMP8 degrades components of the ECM, thus altering the ECS, and neuronal activity and ultimately promoting depression-like behavior. Created with BioRender.com, license agreement VF26OEUBPH

Regarding pharmacological interventions, it will be interesting to decipher which elements of the intricate mechanism regulating MMP8’s enzymatic activity might be affected by CSDS. Given the availability of chemical MMP8 inhibitors, some of them in clinical trials,^4^ their usefulness in depression treatment should be considered. Of cautionary note, MMP8 has multiple targets including non-matrix components relevant to neuronal and non-neuronal cells. Furthermore, post-mortem studies in psychiatric disorders assessing monocyte attachment and MMP8 abundance in the NAc, including ECS enlargement, are looked for.

Broadly speaking, MDD has a highly polygenic genetic architecture where each patient harbors an almost unique portfolio of risk variants.^5^ This poses an extraordinary challenge to dissect out single, causative factors. By contrast, preclinical studies like this report^1^ provide well-defined paradigms of reduced complexity with the opportunity to directly access the role of candidate factors in the living brain. Even if MMP8’s final role in MDD is left to future studies, its expression changes in monocytes could serve as biomarkers in patient stratification and for tailored treatments with novel drugs targeting Mmp8 directly, or pathways leading to Mmp8 expression and activation.

## References

[1] Cathomas, F. et al. Circulating myeloid-derived MMP8 in stress susceptibility and depression. *Nature* **626**, 1108–1115 (2024). 38326622 10.1038/s41586-023-07015-2 PMC10901735

[2] GBD 2017 DALYs and HALE Collaborators. Global, regional, and national disability-adjusted life-years (DALYs) for 359 diseases and injuries and healthy life expectancy (HALE) for 195 countries and territories, 1990–2017: a systematic analysis for the Global Burden of Disease Study 2017. *Lancet* **392**, 1859–1922 (2018). 30415748 10.1016/S0140-6736(18)32335-3 PMC6252083

[3] Beurel, E., Toups, M. & Nemeroff, C. B. The bidirectional relationship of depression and inflammation: double trouble. *Neuron* **107**, 234–256 (2020). 32553197 10.1016/j.neuron.2020.06.002 PMC7381373

[4] Baidya, S. K., Banerjee, S., Guti, S., Jha, T. & Adhikari, N. Matrix metalloproteinase-8 (MMP8) and its inhibitors: a minireview. *Eur. J. Med. Chem. Rep.* **10**, 100130 (2024).

[5] Gordon, J. A. & Binder, E. B. (eds.) *Exploring and Exploiting Genetic Risk for Psychiatric Disorders* (The MIT Press Cambridge, 2023). 39874400

